# Intraperitoneal Hemorrhage in a Pregnant Woman with Hyperemesis Gravidarum: Vitamin K Deficiency as a Possible Cause

**DOI:** 10.1155/2016/5384943

**Published:** 2016-08-11

**Authors:** Yosuke Baba, Hiroyuki Morisawa, Koyomi Saito, Hironori Takahashi, Kazuma Rifu, Shigeki Matsubara

**Affiliations:** ^1^Department of Obstetrics and Gynecology, Jichi Medical University, Tochigi 09216, Japan; ^2^Department of Surgery, Jichi Medical University, Tochigi 09216, Japan

## Abstract

Hyperemesis gravidarum can cause various vitamin deficiencies. Vitamin K deficiency can lead to coagulopathy or hemorrhagic diathesis. A nulliparous Japanese woman with hyperemesis gravidarum at 10^5/7^ weeks was admitted with giant myoma, intestinal obstruction, and abdominal pain. Treatment for a degenerative myoma was instituted with intravenous antibiotics. The abdominal pain ameliorated, but intestinal obstruction persisted. At 16^6/7^ weeks, we performed laparotomy for release of intestinal obstruction, when intraabdominal bleeding of 110 mL existed. Blood tests revealed coagulopathy secondary to vitamin K deficiency. The coagulopathy responded to intravenous vitamin K injection. Coagulopathy due to vitamin K deficiency can occur with hyperemesis gravidarum, and coexisting intestinal obstruction and broad-spectrum antibiotics can aggravate the deficiency.

## 1. Introduction

Hyperemesis gravidarum (HG) can cause maternal-fetal coagulopathy due to vitamin K (VK) deficiency. Of them, neonatal hemorrhage and VK-deficiency embryopathy-fetopathy have been paid attention to [1–4]. Here, we describe intraperitoneal hemorrhage in a pregnant woman with HG. Coexisting intestinal obstruction and antibiotics administration may have exaggerated the VK deficiency and, thus, may have made maternal coagulopathy manifesting.

## 2. Case Report

**Figure 1:**
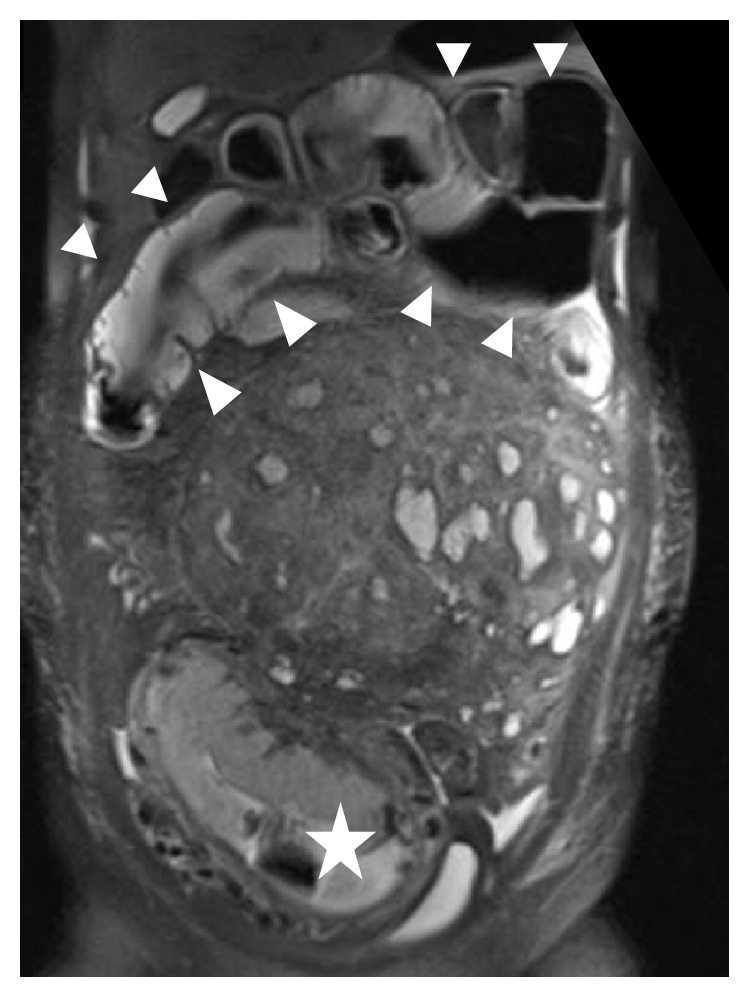
Magnetic resonance imaging of this patient at 12^6/7^ weeks of gestation. Coronal T2-weighted image shows the uterus (star). A large myoma and a dilated intestinal tract (arrowhead) are visible.

In a 36-year-old nulliparous woman with no family history of coagulopathy (62.0 kg and 155.0 cm), ultrasound revealed a giant uterine myoma. At 10^5/7^ weeks, she suffered severe nausea and vomiting. Intravenous electrolyte and fluid administration with thiamine did not ameliorate her condition. At 12^6/7^ weeks, she had lost 6.0 kg in bodyweight with abdominal pain and no defecation: she was hospitalized for HG and suspected intestinal obstruction: laboratory data showed white blood cells, 9500/*µ*L; hemoglobin, 13.8 mg/dL; hematocrit, 42.8%; and C-reactive protein (CRP), 18.0 mg/dL. Transvaginal ultrasound revealed normally growing fetus (crown rump length: 54 mm), and fluid accumulation in the Douglas poach was not evident. Magnetic resonance imaging revealed a giant subserosal myoma and dilated bowel (Figure 1). Diagnosing this condition as HG + degenerated or (partly) infected myoma + intestinal obstruction, fluid replacement and antibiotic (cefmetazole) administration were started, which partly ameliorated her condition. However, defecation did not resume and she lost a further 2 kg (54 kg): we decided on laparotomy at 16^6/7^ weeks.

Intraperitoneal hemorrhage of 110 mL was noted. Myoma adhered to the small intestinal mesenterium, which was considered to be the cause of the intestinal obstruction. Adhesiolysis was performed. It was the experienced surgeon's impression that bleeding occurred from the adhesiolysis surface much more markedly than expected based on the status of adhesion. Although the surface of the pedunculated myoma looked a little ischemic and oozing occurred from the myoma surface on very light touch, bleeding occurred mainly from the mesenterial side (intestinal side) rather than the uterine side at the adhesiolysis. Thus, also considering the presence of intraperitoneal hemorrhage, we suspected the presence of a general hemorrhagic tendency. We resected the pedunculated myoma with total blood loss of 290 mL. Histology confirmed leiomyoma with some foci of degeneration and infection. The postoperative laboratory data suggested coagulopathy: prothrombin time: 14.2 seconds (normal: 10.4–12.2) and PT-International Normalized Ratio (INR): 1.24. The activated partial thromboplastin time (APTT) remained normal (32.5 sec). The intravenous administration of vitamin K (10 mg/day) immediately normalized the PT. At 18^0/1^ weeks, food intake became possible. She vaginally gave birth to an infant, who showed no signs of VK-deficiency-related embryopathy-fetopathy. The mother and infant showed uneventful courses.

## 3. Discussion

HG can cause VK deficiency, leading to maternal hemorrhagic tendency. In this patient hemorrhagic tendency was manifested as intraperitoneal hemorrhage and bleeding at adhesiolysis, which was observed during the surgery.

VK is a cofactor of various coagulation factors, especially Factor VII. A fall in Factor VII causes a prolonged PT, with a normal APTT. Prolongation of PT has already been shown in some HG patients [1, 4, 5]. VK administration rapidly ameliorated the prolonged PT in this patient. All these findings strongly suggested that VK deficiency caused both the observed laboratory coagulation disorder and clinically evident hemorrhagic diathesis.

Reports showed that HG caused maternal and, thus, fetal/neonatal, VK-deficiency-related coagulopathy. Of them, fetal/neonatal brain hemorrhage [1, 2] and VK-deficiency-embryopathy [3] were reported and, thus, have been paid attention to. Few reports showed that maternal hemorrhage was the first manifestation in HG-related VK deficiency. Robinson et al. [6] and Devignes et al. [7] reported that VK-deficiency-related coagulopathy in HG caused marked maternal epistaxis and maternal skin/mucosa hemorrhage, respectively. The present report also showed that maternal hemorrhage or a hemorrhagic tendency can be the first manifestation of this condition.

Women with HG can lack both types of VK required for coagulation, phylloquinone (VK1) and menaquinone (VK2). The former, abundant in leafy vegetables, is obtained from the diet, and the latter is synthesized by ileal flora. Both VK types are absorbed through the small intestine in the presence of bile. The broad-spectrum antibiotic cefmetazole may have eradicated the normal bacterial flora, which normally produce VK2. In fact, inadequate dietary intake and broad-spectrum antibiotic use can cause VK deficiency [8]: this phenomenon is known among internists; however, symptoms/signs usually manifest in older-aged patients, in whom there are limited VK reserve and gastrointestinal malabsorption. In this patient, the following four overlapped: inadequate dietary intake due to HG, intestinal malabsorption due to intestinal obstruction, bile secretory failure at HG [9], and antibiotic use: all these, overlapping altogether, may have caused this condition.

HG, via VK deficiency, can cause maternal hemorrhagic diathesis, especially in patients with intestinal obstruction and taking broad-spectrum antibiotics. Checking the PT may facilitate the earlier detection of this condition. Although this was not measured in this patient, protein induced by vitamin K absence or antagonist- (PIVKA-) II, a more sensitive marker than PT or PT-INR and is preferred over direct VK measurement, may reveal earlier detection of VK-deficiency, and, thus, its measurement may be considered in patients with HG, especially at the time of surgery. Robinson et al. [6], who reported HG-related VK-deficiency-induced maternal hemorrhagic diathesis (epistaxis), recommended that “prophylactic VK replacement should be considered in cases in which HG is severe and protracted.” Another report also supported this view [4]. Although this sounds reasonable, further studies are necessary to determine whether universal prophylactic administration of VK in severe HG patients should be performed.

## Abbreviations

APTT: Activated partial thromboplastin time
CRP: C-Reactive protein
HG: Hyperemesis gravidarum
INR: International Normalized Ratio
PIVKA: Protein induced by vitamin K absence or antagonist
PT: Prothrombin time
VK: Vitamin K.
